# Suicide risk genes impact evolutionarily conserved survival strategies

**DOI:** 10.21203/rs.3.rs-7800765/v1

**Published:** 2025-10-24

**Authors:** Alexa Dustin, Donard Dwyer

**Affiliations:** LSU Health Shreveport

## Abstract

Genome-wide association studies (GWAS) and candidate gene analyses have identified possible suicide risk genes that are highly conserved during evolution and enriched in genes essential for life. However, functional roles for these risk genes have not been confirmed and pathways from risk variant to relevant phenotype to suicidality-related behavior remain unknown, highlighting critical gaps in our knowledge. Here, we report findings from the largest behavioral and mechanistic study of suicide risk genes to date. In *Caenorhabditis elegans*, mutations in risk gene counterparts caused exaggerated threat evaluation (social feeding) and diminished motivation to seek food, which represent ancient strategies for avoiding harm and ensuring survival (foraging). Genetic variation affected neuropeptide (NPY and TGF-b) function and kinase signaling. Remarkably, the altered behaviors were corrected with drugs that reduce suicidal behavior including antidepressants and clozapine. Taken together, these findings reveal that risk genes predisposing a person to take their life normally promote strategies to survive.

## Introduction

The World Health Organization reports nearly 800,000 deaths by suicide every year [1]. In addition, many times this number of individuals (~10 million in the United States) experience suicidal thoughts and behaviors reflecting a significant population in severe distress [2]. Although suicide results from many factors, unaddressed mental health issues such as depression and feelings of hopelessness, constant existential threat or stress, and loss of motivation for engagement in life are major contributors [3–8]. Furthermore, suicide often occurs within families, which is consistent with a strong genetic basis for suicidality-related behavior such as suicidal thoughts and attempted suicide [9–12]. Recently, a list of 105 putative suicidality-related risk genes was compiled from genome-wide association studies (GWAS), whole exome sequencing and candidate gene analyses [13]. The risk genes were highly conserved through evolution and were enriched in genes essential for life [13]. Major questions remain concerning these proposed genetic risk factors, for example: 1) is there functional evidence that supports their establishment as bona fide suicide risk genes and 2) what do these putative risk genes do? Answers to these questions would strengthen our confidence in the findings of genetic association studies and may provide insights into the underlying processes that predispose someone to suicidal behavior.

Suicide risk genes are unlikely to specify the full spectrum of behaviors associated with suicidality; rather, they may produce endophenotypes such as hypothalamus-pituitary-adrenal (HPA) axis dysfunction, altered serotonergic activity, diminished motivation and exaggerated threat assessment [8, 14–16]. We have identified two evolutionarily conserved counterparts in *Caenorhabditis elegans* of these endophenotypes – termed protophenotypes [17] – namely, reduced goal-directed foraging (diminished motivation) and social feeding. A major goal of these studies is to determine whether mutations in putative suicide risk genes can produce these protophenotypes in *C. elegans* because most of the risk genes have been evolutionarily conserved. If so, it would lend support for their role in suicidality-related behavior and allow us to characterize the pathways involved.

Social feeding in *C. elegans* is defined by worms preferring to gather at the edge of a bacterial lawn, known as bordering, and then aggregating into discrete clumps [18]. It contrasts with solitary feeding where animals individually disperse throughout the lawn of bacteria. Wild isolates of *C. elegans* tend to exhibit social feeding; however, solitary feeding strains are also prevalent [19]. Although described as social behavior, the aggregation of *C. elegans* on bacteria is actually a compensatory response to sensing elevated O_2_ levels in the context of food signals and population density [20]. Social feeding strains sense ambient O_2_ levels as aversive, which promotes initial clustering at locations where levels are lower such as the border of the bacterial lawn [20, 21]. O_2_ is further reduced by aggregation during social feeding [22], thereby attenuating the perception of adverse conditions. Thus, social feeding reduces aversive signaling – a ‘safety in numbers’ strategy used widely in nature. Nevertheless, the perception of ambient O_2_ levels as harmful should be considered **exaggerated** threat detection because the actual risk is negligible. Stated differently, social feeding mutants respond with excessive measures - bordering, swarming and aggregation - to conditions that offer no real threat. In fact, social feeding strains such as *npr-1* live longer than wild-type N2 animals [23].

The N2 Bristol strain, which is widely used as the reference or standard for comparison, is a solitary feeding strain. By contrast, reduction-of-function and null mutations in *npr-1*, an ortholog of the mammalian neuropeptide Y receptor gene, produce social feeding phenotypes on the N2 background [18]. However, social feeding is not limited to mutants with variation in *npr-1*; mutations in additional genes, for example *daf-7*, *glb-5, exp-1* and *pdk-1*, are associated with bordering on food and aggregation [24–26]. -

Interestingly, low levels of neuropeptide Y (NPY) have been implicated as a suicide risk factor [27, 28], while genetic analysis has also implicated NPY [29]. These findings are consistent with the physiological role of NPY as an anxiolytic neuropeptide [30, 31] that regulates threat and feeding responses [32]. PDPK1 (*pdk-1*) or 3-phosphoinositide dependent kinase 1, represents an additional suicide risk gene [33] that has been reported to cause social feeding in *C. elegans* [25], although this response has not been further characterized. In addition, the role of *daf-7* in social feeding is worth investigation because a human counterpart, transforming growth factor-b (TGF-b), has been implicated in suicide [34, 35]. Consequently, we sought to evaluate whether additional *C. elegans* alleles recently identified as counterparts of human suicide risk genes [13] caused social feeding and diminished motivation phenotypes. -

In previous studies of *npr-1* mutants, we demonstrated that antipsychotic drugs and calmodulin antagonists converted social feeding to solitary feeding [36]. Based on these findings, we hypothesized that drugs shown clinically to reduce suicidal behavior might reverse the social feeding phenotype. Here, we report that loss-of-function mutations in 6 of 19 suicide risk genes promoted social feeding that was reversed with certain antipsychotic drugs, antidepressants and serotonin receptor antagonists. Furthermore, loss-of-function mutations in the NPY receptor (*npr-1*) and TGF-b counterpart (*daf-7*) produced diminished motivation in a foraging assay akin to apathy or hopelessness. We have derived neuronal networks and molecular pathways responsible for risk gene effects on relevant protophenotypes. These findings provide new insights into the function of putative suicidality-related risk genes and suggest a unifying theme for their activity – promoting survival of the organism.

## Materials and Methods

### Strains and routine culture

All *C. elegans* strains were obtained from the Caenorhabditis Genetics Center at the University of Minnesota except QL300 (*tph-1;daf-7*), which was kindly provided by the QueeLim Ch’ng laboratory [37]. The strains are listed in Table 1 together with gene designations and a brief summary of their evaluation in a separate experimental model related to suicidal behavior [13]. Several *npr-1* social feeding strains were evaluated in these studies along with 16 strains with loss-of-function mutations in putative suicide risk genes [13]. Furthermore, *daf-7* mutants were investigated because the *e1372* allele has been reported to promote social feeding and TGF-b has been linked to suicide risk. The wild-type Bristol variant N2 strain served as the standard for comparison. All strains were cultured at 20 °C according to general methods described by Brenner [38]. For these studies, the animals were grown on large (100 mm diameter) nematode growth medium (NGM) agar plates with 5X peptone and a large bacterial lawn to obtain sufficient numbers of adult animals for the social feeding assay.

### Drugs

The drugs used in this study were purchased from Millipore-Sigma-Aldrich or Bio-Techne Corp. They were dissolved in dimethyl sulfoxide (DMSO) to obtain 40 mM stock solutions that were then diluted in dilute (1.7 mM) acetic acid before adding to the surface of agar plates.

Drugs were generally tested at a final concentration on the plates of 160 mM, which was selected based on numerous previously published studies [39, 40–43]. Lithium was evaluated at 8.8 mM, which was similar to other reports [41, 44]. Lithium is not toxic at this concentration – it extended lifespan in *C. elegans* at 10 mM [41]. Several groups have shown that drug concentrations achieved in the worm via drug exposure on plates is about one-hundredth of that applied to the plate [41, 45, 46]. Although the affinity of these drugs for *C. elegans* neurotransmitter receptors is in the range of 25–100 nM in direct binding assays [47, 48], higher concentrations (50–200 mM) are required on the plate to produce physiological effects in vivo mediated by these same receptors [40–43, 45]. This difference may result from restricted uptake through the cuticle and distribution issues.

### Social feeding assay

Social feeding was assessed by adapting a previously reported method [36]. First, we prepared small plates that were coated with 200 ml of control buffer (dilute acetic acid plus DMSO equal to the volume of the drugs tested) or drug dissolved in DMSO and dilute acetic acid for 2 hr prior to use. Briefly, animals were harvested from a large 5X peptone agar plate in ~1.5 ml of M9 buffer, which was pipetted into an Eppendorf tube. They were allowed to gently settle to the bottom over several minutes and the supernatant was then removed leaving a volume sufficient to add to the desired number of small plates – generally 4–8 assay plates per large growth plate. Animals were resuspended by flicking the tube and 20 ml of the suspension was added to the test plates in 8 spots evenly distributed around the circular bacterial lawn. The spots were located about 5 mm away from the lawn so that animals had to navigate their way to food. Plates were put in an incubator at 20 °C and examined for aggregation after 2, 4 and 5 hr. Clumps of animals > 0.5 mm across and on the border of the bacterial lawn were counted as aggregates. We could not control for the precise number of worms harvested from the plates during different repeats of the experiment, which led to variable numbers of aggregates between trials for the same mutant strain, for example 20 in experiment A vs. 7 in experiment B. Therefore, it was necessary to use a matched sample design where pairs of plates were set up in each iteration of the experiment for the purpose of statistical comparison, e.g., *npr-1(ky13)*-DMSO vs. *npr-1(ky13)*-drug, where the same source of worms was used for drug vs. DMSO conditions.

### Immobility assay of diminished goal-directed behavior

Previously, we have described assessment of motivation to search for food following transfer to plates lacking bacteria [39]. Typically, animals show greater overall activity and characteristic searching behavior upon acute food deprivation, whereas mutants with defects in insulin signaling or in different putative suicidality-related genes show decreased activity and become immobile on plates without bacteria [13, 39]. The assay was performed at 25 °C throughout. At this temperature, animals are much more active, which accentuates the immobility phenotype. Briefly, for this assay, animals (20–25) were incubated on small plates with food and DMSO (control) or drug for 2 hr. They were then transferred to large plates (100 mm diameter) without bacteria but containing DMSO or drug to match the initial plate and were assessed for movement after 2.5 and 20 hr. Animals were only counted as immobile if they were not moving but rapidly escaped touch to the tail, which confirmed capability for locomotion. We counted the number of animals moving and the total number on the plate and the data were expressed as the percentage moving. Each experiment was repeated on more than 5 occasions for consistency.

### Analysis of risk gene co-expression

To determine if the suicide risk genes evaluated in this study are co-expressed in various human tissues, we analyzed Co-expression with GeneMANIA [49]. The 6 main risk genes from this work were evaluated along with two others from a previous study (GRIA1 and STK33), the NPY receptor (NPY1R) and a paralog of HIPK2, HIPK3. Briefly, we entered gene names in the search window, toggled Co-expression only, and set the parameters, Max Resultant Genes and Max Resultant Attributes, to 0. For comparison, we generated 20 similar-sized lists of random genes obtained from MolBiotools and analyzed these for Co-expression. We obtained images showing networks of genes that are co-expressed. Hub genes that connected with at least 3 other genes were counted as a measure of network connectivity. Data from the 20 random gene sets were averaged and the standard deviation was calculated to obtain a confidence interval at the p = 0.05 level.

To examine co-expression of suicide risk genes in the human brain, we analyzed expression heat maps and the corresponding quantitative data derived from the Allen Human Brain Atlas website [50] for the same 10 risk genes evaluated in GeneMANIA. We focused on brain areas where 6 or more of the genes were co-expressed as judged by z-scores > 1. A total of 20 unique brain areas with high co-expression of the suicide risk genes was identified in this analysis.

### Statistics

In the social feeding assay, each small test plate represented an individual data point and experiments were repeated over 5 or more separate trials to obtain sufficient data for analysis. Because the total number of worms harvested from the large growth plates varied between trials, which affected the number of aggregates observed, we used a matched sample design for this study to account for this variability. A paired t-test is the standard for statistical comparison of matched sample data. Each pair for analysis consisted of data from the control group compared with data from a particular drug concentration collected in the same trial with the same source of animals. In initial studies, we compared aggregation of the strains with mutations in putative suicide risk genes with data obtained using N2 animals. A standard t-test was used for these comparisons.

Data from the immobility assay were analyzed with a chi square-test as described previously [39] due to the non-parametric nature of the readout - moving vs. not moving. We collected data from a minimum of 5 experiments that encompassed two time points, 2.5 and 20 hr. In the figures, we expressed the data as averages of % Moving calculated from all repeats of the experiment. Standard errors are not generated with this analysis; however, we have included the total number of animals from the pooled set of data. We compared N2 vs. mutant and mutant-DMSO vs. mutant-drug groups. All p values < 0.05 were considered significant.

## Results

### Social feeding in suicidality-related risk gene mutants

**Figure 1:**
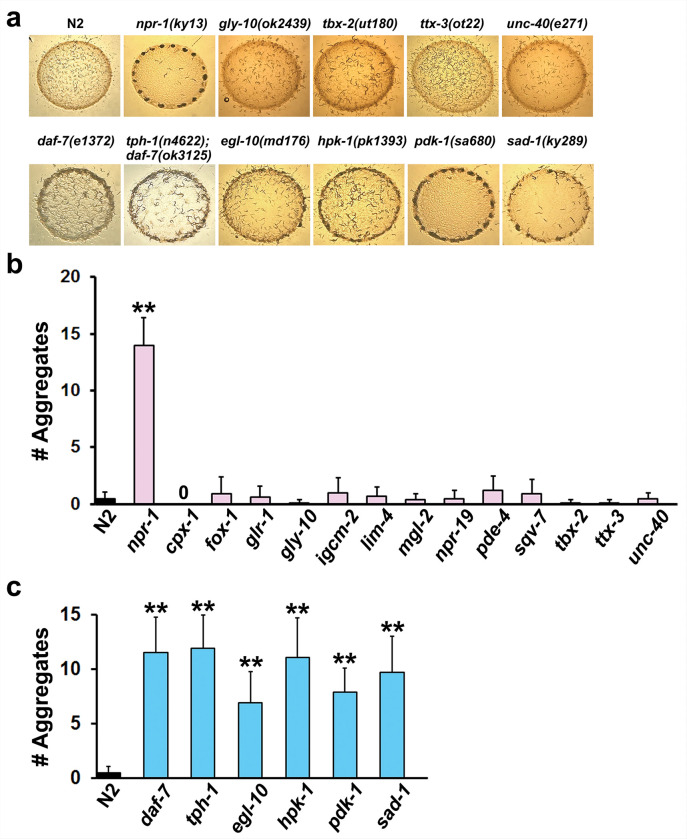
Social feeding in strains with mutations in suicidality-related risk genes. **a)** Photographs of wild-type controls (N2), *npr-1(ky13)* (positive control), representative negative mutants (labeled, upper panel right) and social feeding mutants (lower panel). **b & c)** Quantitation of aggregates formed at the border of the bacterial lawn for each of the mutants. Averages of multiple independent experiments are shown here, and the error bars represent standard deviations. The zeroes on the x-axis in this figure and others indicate that no aggregates were observed. In 1c, *tph-1* refers to the *tph-1;daf-7*double mutant. Asterisks indicate significant differences (**p < 0.01) when compared to the N2 controls. The statistics for each social feeding mutant compared with N2s: *npr-1* [df 24, t-stat 20.9, p=6.4E-17], *daf-7* [df 18, t-stat 10.2, p=6.6E-09], *tph-1;daf-7* [df 22, t-stat 10.5, p=4.9E-10], *egl-10*[df 18, t-stat 6.5, p=3.7E-06], *hpk-1* [df 18, t-stat 9.0, p=4.1E-08], *pdk-1*[df 14, t-stat 9.1, p=3.0E-07] and *sad-1* [df 18, t-stat 8.3, p=1.5E-07].

Based on the fact that two genes linked to social feeding (*npr-1* and *daf-7*) are also implicated as suicide risk factors, we reasoned that additional suicidality-related risk genes may cause social feeding phenotypes in *C. elegans*. Therefore, we evaluated whether mutations in 17 previously characterized suicide risk-gene counterparts produced social feeding – bordering, clumping and stable aggregation. We also examined 5 mutants related to the *npr-1* and *daf-7* genes in this study (see Table 1 for the complete list of strains). Most of the 17 mutants (13) were solitary feeders like the N2 wild-type animals (Fig. 1). However, 4 of the strains with mutations in suicidality-related risk genes exhibited social feeding (Fig. 1a & 1c). This finding nearly doubles the total number of social feeding strains identified to date, which highlights the overall rarity of mutations that promote social feeding [26].

Although the 7 mutants depicted in Fig. 1b & 1c all displayed bordering and aggregation typical of social feeding, there were subtle differences that are partially captured in Fig. 1a. *daf-7* and *egl-10* mutants were similar and showed a swarming phenotype at the bacterial border rather than tight aggregation as observed with *npr-1* mutants. Individual clumps could be identified, but their structure was more diffuse and less stacked in three dimensions. In addition, aggregates were more dynamic in the *tph-1;daf-7* double mutants that lack serotonin. Despite these subtle differences, the fact that 6 different suicide risk genes of 19 tested were associated with social feeding was remarkable and merited further investigation.

### Effect of antidepressants, antipsychotics and lithium on social feeding induced by loss-of-function mutations in suicide risk-gene counterparts

**Figure 2:**
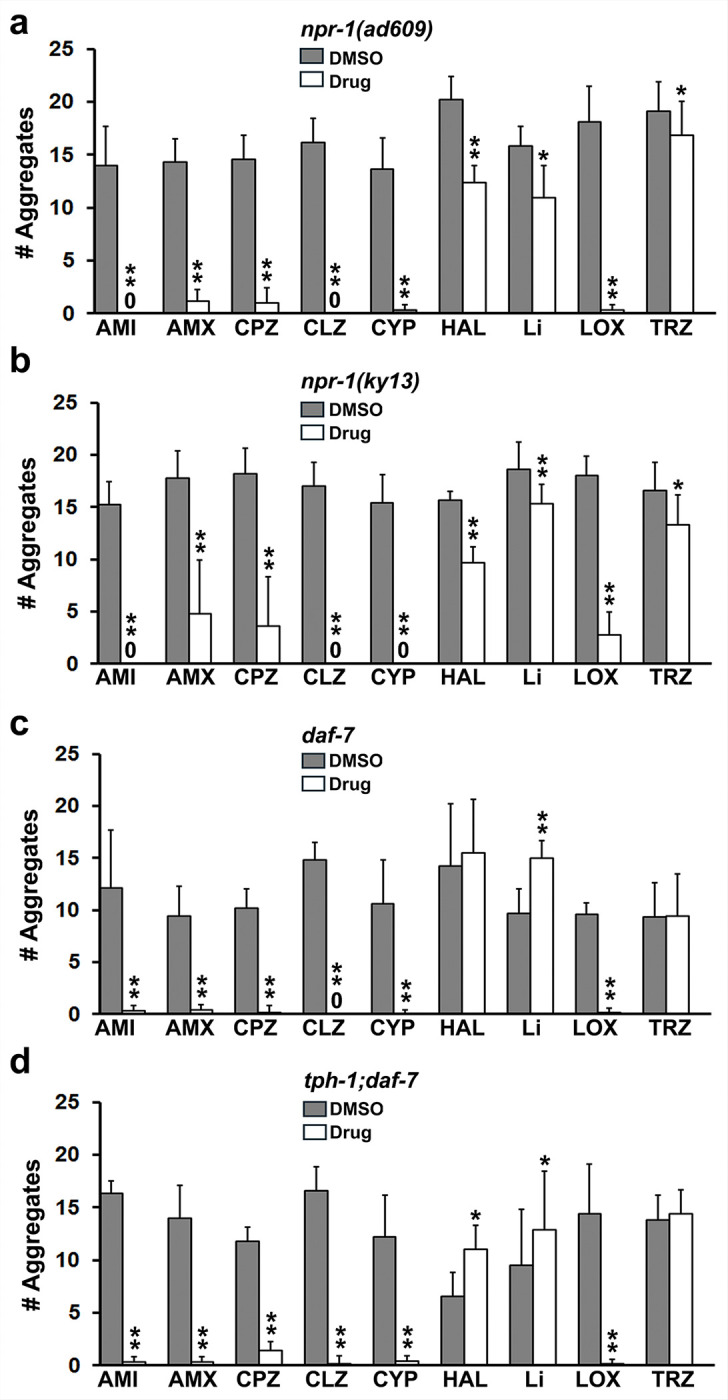
Effect of psychotropic drugs on social feeding in established strains. **a) *npr-1(ad609)***. Social feeding was measured in the absence (DMSO control) or presence of drugs at 160 mM, except for lithium present at 8.8 mM. These data were obtained 4–5 hr after the addition of animals to the plates. For statistical purposes, we compared the drug and DMSO groups from each experiment and the data are expressed as the averages plus the standard deviations pooled from multiple experiments. Asterisks indicate significant differences in a-d: *p < 0.05, **p < 0.01. Drugs are abbreviated as follows: amitriptyline-AMI, amoxapine-AMX, chlorpromazine-CPZ, clozapine-CLZ, cyproheptadine-CYP, haloperidol-HAL, lithium-Li, loxapine-LOX and trazodone-TRZ. Statistics: AMI [df 4, t-stat 15.7, p=9.7E-05], AMX [df 4, t-stat 9.4, p=0.0007] CPZ [df 9, t-stat 10.1, p=3.2E-06], CLZ [df 4, t-stat 16.2, p=8.5E-05] CYP [df 4, t-stat 12.7, p=0.0002], HAL [df 5, t-stat 10.4, p=0.0001], Li [df 7, t-stat 2.9, p=0.02], LOX [df 7, t-stat 14.3, p=1.9E-06] and TRZ [ df 8, t-stat 2.8, p=0.02]. **b) *npr-1(ky13)***. Statistics: AMI [df 4, t-stat 8.3, p=0.001], AMX [df 5, t-stat 11.0, p=0.0001], CPZ [df 4, t-stat 13.2, p=0.0002], CLZ [df 4, t-stat 16.7, p=7.5E-05], CYP [df 6, t-stat 10.6, p=4.8E-05], HAL [df 4, t-stat 13.4, p=0.0002], Li [df 7, t-stat 6.2, p=0.0004], LOX [df 8, t-stat 15.3, p=3.3E-07], and TRZ [df 8, t-stat 4.1, p=0.003]. **c) *daf-7***. Statistics: AMI [df 7, t-stat 6.0, p=0.0005], AMX [df 4, t-stat 6.4, p=0.003], CPZ [df 9, t-stat 16.3, p=5.5E-08], CLZ [df 5, t-stat 21.1, p=4.4E-06], CYP [df 8, t-stat 7.3, p=8.2E-05], HAL [df 7, t-stat −1.1, p=0.32], Li [df 5, t-stat −6.3, p=0.001], LOX [df 4, t-stat 23.5, p=1.9E-05], TRZ [df 8, t-stat −0.1, p=0.92}. ***d) tph-1;daf-7***. Statistics: AMI [df 5, t-stat 27.7, p=1.1E-06], AMX [df 5, t-stat 10.6, p=0.0001], CPZ [df 4, t-stat 12.8, p=0.0002], CLZ [df 8, t-stat 20.4, p=3,4E-08], CYP [df 4, t-stat 6.6, p=0.002], HAL [df 4, t-stat −2.8, p=0.04], Li [df 7, t-stat −3.3, p=0.01], LOX [df 4, t-stat 6.8, p=0.002] and TRZ [df 4, t-stat −0.6, p=0.55].

Drugs that reduce depression, anxiety and agitation were previously shown to decrease social feeding [36], which is mediated by exaggerated threat assessment systems. Therefore, we examined a panel of psychotropic drugs for their ability to inhibit social feeding in the newly identified mutant strains. As the starting point, we determined that different drugs from various classes significantly inhibited social feeding in both *npr-1* mutants with similar profiles (Fig. 2). Some were very potent, e.g., amitriptyline (AMI), chlorpromazine (CPZ), clozapine (CLZ) and cyproheptadine (CYP, a 5-HT2 receptor antagonist), while others produced less inhibition, e.g., lithium (Li) and trazodone (TRZ). Although many of the drugs likewise prevented social feeding in the *daf-7* mutants, there were notable differences from the *npr-1* findings (Fig. 2c & 2d). Trazodone did not affect aggregation, whereas lithium **promoted** formation of aggregates in these two strains. Furthermore, haloperidol failed to inhibit social feeding in *daf-7* and modestly, but significantly increased aggregation in *tph-1;daf-7* double mutants. Interestingly, the inhibitory actions of cyproheptadine in the *tph-1;daf-7* mutant (Fig. 2d), which lacks serotonin, suggest involvement of additional targets such as calmodulin [36]. These data and others to follow rule out non-specific effects of the drugs on social feeding.

**Figure 3:**
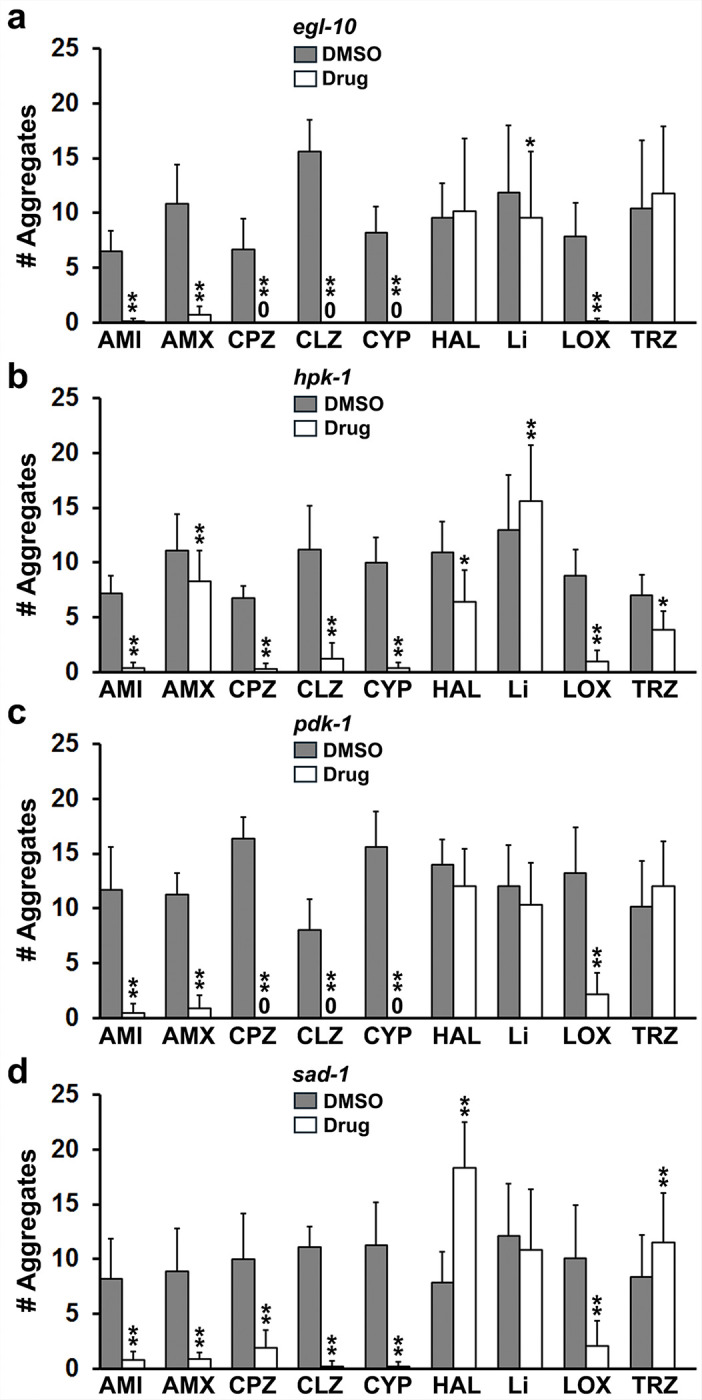
Psychotropic drugs affect aggregation in newly-characterized social feeding mutants. The data in these graphs were obtained for the 4 mutants as described in the legend to Fig. 2. **a) *egl-10*.** Statistics: AMI [df 24, t-stat 12.0, p=1.9E-11], AMX [df 5, t-stat 6.9, p=0.0009], CPZ [df 5, t-stat 5.8, p=0.002], CLZ [df 4, t-stat 11.7, p=0.0003], CYP [df 5, t-stat 8.3, p=0.0004], HAL [df 11, t-stat −0.4, p=0.7], Li [df 7, t-stat 4.6, p=0.002], LOX [df 7, t-stat 6.8, p=0.0002] and TRZ [df 6, t-stat −1.2, p=0.27]. **b) *hpk-1***. Statistics: AMI [df 4, t-stat 7.9, p=0.001], AMX [df 9, t-stat 7.4, p=4.0E-05], CPZ [df 5, t-stat 15.2, p=2.2E-05], CLZ [df 5, t-stat 6.1, p=0.002], CYP [df 4, t-stat 10.3, p=0.0005], HAL [df 6, t-stat 3.1, p=0.02], Li [df 7, t-stat −8.1, p=8.4E-05], LOX [df 4, t-stat 6.7, p=0.002] and TRZ [df 7, t-stat 3.0, p=0.02]. **c) *pdk-1***. Statistics: AMI [df 5, t-stat 6.8, p=0.001], AMX [df 6, t-stat 18.2, p=1.7E-06], CPZ [df 7, t-stat 24.1, p=5.4E-08], CLZ [df 4, t-stat 6.3, p=0.003], CYP [df 6, t-stat 12.6, p=1.5E-05], HAL [df 4, t-stat 2.4, p=0.07], Li [df 6, t-stat 1.9, p=0.09], LOX [df 9, t-stat 11.7, p=9.1E-07] and TRZ [df 10, t-stat −2.1, p=0.07]. **d) *sad-1***. Statistics: AMI [df 4, t-stat 4.4, p=0.01], AMX [df 7, t-stat 5.8, p=0.0006], CPZ [df 6, t-stat 7.4, p=0.0003], CLZ [df 7, t-stat 16.3, p=7.9E-07], CYP [df 5, t-stat 71., p=0.0009], HAL [df 7, t-stat 3.0, p=0.02], Li [df 9, t-stat 1.7, p=0.13], LOX [df 7, t-stat 6.4, p=0.0004] and TRZ [df 7, t-stat −5.4, p=0.001].

Figure 3 summarizes the results with the 4 new social feeding mutants characterized here. Tricyclic antidepressants, atypical or second-generation antipsychotic drugs (clozapine and loxapine, LOX), chlorpromazine and cyproheptadine significantly inhibited aggregation in all 4 mutants. Haloperidol did not affect social feeding in *egl-10* and *pdk-1* mutants, reduced it in *hpk-1* and significantly increased aggregate formation in *sad-1* mutants. Lithium was negative in *pdk-1* and *sad-1* mutants and modestly inhibited or promoted social feeding in *egl-10* and *hpk-1* mutants, respectively. Trazodone significantly reduced aggregation in *hpk-1* mutants and significantly enhanced social feeding in *sad-1* mutants. Taken together, the data demonstrate that the aversive signaling that mediates social feeding can be modified pharmacologically by drugs that restore a sense of wellbeing in patients with psychiatric disorders – this did not have to be the case. The different effects of haloperidol, lithium and trazodone on the mutants indicates selective rather than non-specific effects of these drugs that depend on genetic background. Finally, representatives of the main drug classes did not affect locomotion in N2 animals as judged by body bends/min on and off food (On Food: DMSO 13.3 ± 7.7; amitriptyline 15.2 ± 3.3; clozapine 12.1 ± 3.4; and lithium 11.6 ± 5.8; Off Food (30–60 min): DMSO 41.4 ± 7.3; amitriptyline 40.6 ± 4.5; clozapine 39.7 ± 6.3; and lithium 38.1 ± 6.6; N = 10 per group), which argues against general stimulatory effects of the drugs on movement that destabilize aggregates.

### Social feeding mutants display diminished motivation to search for food

In prior studies, we reported that loss-of-function mutations in *egl-10*, *hpk-1*, *pdk-1* and *sad-1* mutants caused an immobility phenotype in *C. elegans* following brief deprivation of bacteria [13]. This behavior was interpreted as diminished motivation to search for food [39] and was corrected with many of the same psychotropic drugs tested here. Therefore, we hypothesized that the *npr-1* and *daf-7* mutants might similarly show diminished motivation to forage in addition to their established social feeding phenotype. *daf-7* showed a significant reduction in foraging movement at the first time-point (2.5 hr) as well as the second at 20 hr. *tph-1;daf-7* double mutants foraged less at 2.5 hr, although it was not statistically significant, whereas the decrease in foraging at 20 hr was. We previously described such a delayed immobility response (an ‘exhaustion’ phenotype?) in *glr-1* mutants with loss-of-function mutations in a *C. elegans* α-amino-3-hydroxy-5-methyl-4-isoxazolepropionic acid (AMPA) glutamate receptor ortholog [13]. It is worth pointing out that at 20 hr, animals were fully capable of movement and responded rapidly to touch on the tail with a vigorous escape response. Therefore, the lack of movement was not due to paralysis or muscle fatigue but a failure to sustain the drive to forage – the normal response to food deprivation.

**Figure 4:**
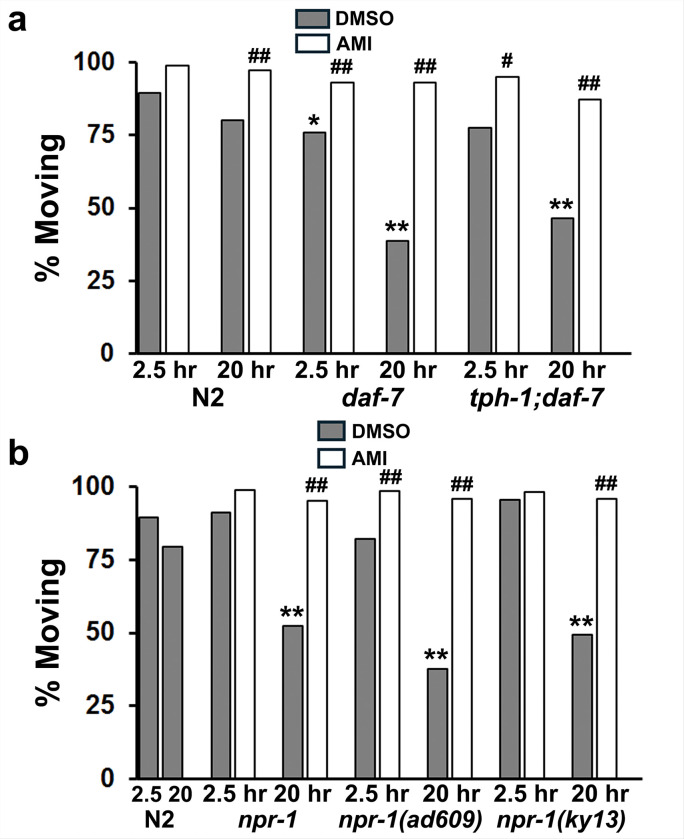
Social feeding strains show diminished motivation to forage. **a)** Social feeding mutants *daf-7* and *tph-1;daf-7* were evaluated for foraging behavior (active searching movement) 2.5 and 20 hr after removal from bacteria. We compared their response (% Moving) to that of N2 animals (control). In addition, we tested whether amitriptyline (AMI, 160 mM) would restore foraging behavior at both time points. Chi-square tests revealed significant differences from N2 controls as indicated with asterisks – *p < 0.05, **p < 0.01 – whereas significant differences between DMSO and AMI have been indicated with hash marks: ^#^p < 0.05, ^##^p < 0.01. ***daf-7*** Statistics: vs. N2 2.5 hr [N=255, p=0.004], vs. N2 20 hr [N=200, p=3.6E-09], DMSO vs. AMI 2.5 hr [N=209, p=0.001], DMSO vs. AMI 20 hr [N=141, p=6.7E-11]. ***tph-1;daf-7*** Statistics: vs. N2 2.5 hr [N=196, p=0.12], vs. N2 20 hr [N=173, p=1.2E-05], DMSO vs. AMI 2.5 hr [N=142, p=0.002], DMSO vs. AMI 20 hr [N=135, p=2.9E-07]. **b)** Several *npr-1* mutants were evaluated as described in (4a). ***npr-1*** Statistics: vs. N2 2.5 hr [N=212, p=0.74], vs. N2 20 hr [N=178, p=0.0002], DMSO vs. AMI 2.5 hr [N=174, p=0.002], DMSO vs. AMI 20 hr [N=142, p=2.8E-09]. ***npr-1(ad609)*** Statistics: vs. N2 2.5 hr [N=252, p=0.09], vs. N2 20 hr [N=215, p=4.2E-10], DMSO vs. AMI 2.5 hr [N=240, p=2.1E-05], DMSO vs. AMI 20 hr [N=193, p=1.4E-17]. ***npr-1(ky13)*** Statistics: vs. N2 2.5 hr [N=223, p=0.11], vs. N2 20 hr [N=186, p=1.9E-05], DMSO vs. AMI 2.5 hr [N=147, p=0.35], DMSO vs. AMI 20 hr [N=118, p=6.8E-08].

A second goal of this assay was to determine whether drug treatment could restore normal foraging. Toward this goal, we used amitriptyline, a tricyclic antidepressant, because of its effectiveness in *C. elegans* [13, 39]. As seen in Fig. 4, amitriptyline completely restored goal-directed movement at both time points, which further argues against paralysis or muscle fatigue as explanations for the immobility observed after 20 hr.

When several different *npr-1* mutants were evaluated, we found that they searched for food as usual at 2.5 hr but showed significant reduction in foraging and dispersal activity at 20 hr compared to wild-type N2 animals (Fig. 4b). This lack of goal-directed movement was again corrected by amitriptyline in all three strains. Thus, social feeding strains with an exaggerated sense of threat also exhibited diminished motivation to take actions essential for life like finding food. Moreover, it was remarkable that an antidepressant drug used to combat apathy, anhedonia and suicidal thoughts in depressed patients corrected the phenotypes produced by mutation of suicide risk-gene counterparts in *C. elegans*.

### Gene co-expression and neuronal networks

**Figure 5:**
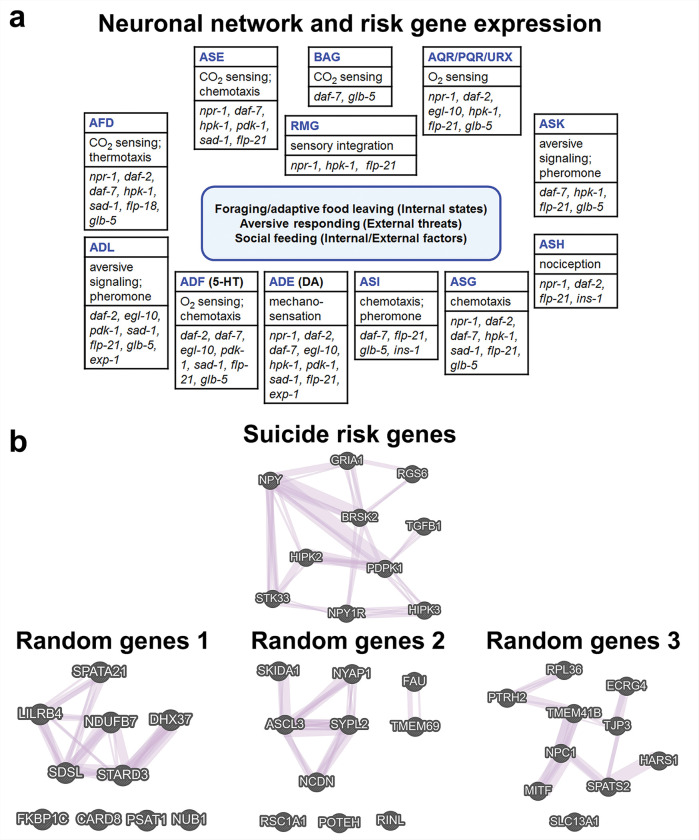
Co-expression of putative suicidality-related risk genes in *C. elegans* (a) and humans (b). **a)** The neurons and networks that mediate social feeding and foraging in *C. elegans* have been mapped previously [51–53] and the main neurons are depicted here as boxes with their 3-letter designation. The major role of each neuron is included along with relevant genes expressed. Most of the neurons use glutamate and neuropeptides for neurotransmission except ADE, which is dopaminergic, ADF, which is serotonergic, and ASI and RMG, which use neuropeptides. **b)** To confirm co-expression of the risk genes in human tissues, we queried the GeneMANIA database. For comparison, we generated 3 similar-sized lists of randomly-selected genes and determined their co-expression profiles (lower panel). Genes depicted individually at the bottom were not co-expressed with any of the other genes in the list.

Because a subset of the suicide risk-gene counterparts was associated with both social feeding and diminished motivation phenotypes in *C. elegans* we would expect to observe co-expression of the genes in neuronal networks responsible for these behaviors. The main neurons involved in O_2_ sensing in social feeding and aversive signaling more broadly, and those that detect food and pheromone signals relevant for foraging and food leaving have been mapped previously [51–53]. Using database information from WormBase and WormAtlas, we constructed the neuronal network depicted in Fig. 5a that highlights expression of the suicide risk gene counterparts. We included the *daf-2* gene (*C. elegans* insulin receptor) and *ins-1* because insulin, along with NPY mediates satiety and motivation to find food [39, 54], *flp-18* and *flp-21*, which are neuropeptide agonists of NPR-1, and *glb-5* and *exp-1* because these genes also regulate social feeding [26]. Several features are notable: 1) there is overlap in the neurons that detect sensory signals to regulate solitary vs. social feeding and satiety/foraging responses, 2) many of the genes contributing to social feeding and motivation to forage are expressed in the same neurons (e.g., the ADE neurons express all the main social feeding genes except *glb-5*), and 3) the neurons are collectively attuned to both external threat signals and internal homeostatic states.

Is there evidence that these same suicide risk genes are also co-expressed in humans? To address this question, we used GeneMANIA to evaluate tissue co-expression of the suicide risk genes studied here along with GRIA1 and STK33 from previous analysis [13]. As can be seen in Fig. 5, the suicide risk genes are highly co-expressed in humans as they are in *C. elegans*. For comparison, we generated similar-sized lists of randomly-selected human genes and analyzed them with GeneMANIA. Although some of the randomly chosen genes are co-expressed in tissues, as would be expected, the level of overlap is less as judged by the number of hub genes co-expressed with at least 3 others. The suicide risk gene set has 7 hub genes vs. 2–4 for the random sets in Fig. 5, which also had genes that were not connected or co-expressed. Analysis of 20 sets of randomly-selected genes revealed an average of 3 hub genes per set of 10 and a 0.05 confidence interval of 0–6.84, which means the suicide risk genes are significantly more networked than random genes.

To further explore whether the suicide risk genes function together, we determined brain areas in the Human Brain Atlas where 6 or more risk genes were co-expressed. The data in Table 2 summarize the findings. Strikingly, the functions of many of the brain areas with high co-expression fit with the story emerging here, namely involvement in sensation of aversive stimuli/conditions (anterior orbital gyrus, spinal trigeminal nucleus), sensory integration (claustrum, pontine nucleus, precuneus, inferior colliculus), threat detection (basomedial/basolateral nuclei, lateral nucleus, bed nucleus stria terminalis) and regulation of motivation and goal-directed behavior (nucleus accumbens, claustrum, basolateral nucleus, putamen). Overall, we conclude that the suicidality-related risk genes are frequently co-expressed in human tissues including brain regions associated with threat evaluation, motivation and reward.

## Discussion

Promising candidates for suicide risk genes have been identified in genetic association studies including GWAS and whole exome sequencing [34, 55, 56]. However, the contribution of these genes to suicide risk has not been confirmed by independent functional analysis nor have relevant phenotypes been characterized. These are serious shortcomings in our understanding of the causal path from risk genes to phenotypes to suicidality-related behavior. To address this major gap in our knowledge, we evaluated 19 unique mutants of *C. elegans* with loss-of-function mutations in suicide risk-gene counterparts to detect changes in two relevant phenotypes: social feeding (threat assessment) and goal-directed behavior (foraging). This approach is justified because suicide risk genes have been highly conserved during evolution, they are expressed in this species, and they are enriched for essential genes that broadly affect behavior [13]. To our knowledge, this is the largest analysis of phenotypes associated with suicide risk genes undertaken to date, and several key findings emerged.

First, we showed that loss-of-function mutations in 6 of 19 suicide risk-gene counterparts produced a social feeding phenotype. A priori, the probability of this outcome was low because mutations that cause social feeding are rare [26]. These findings suggest a significant relationship between suicide risk factors and the behavioral basis of social feeding. Furthermore, drugs that reduce suicidality-related behavior, notably clozapine and antidepressant drugs [57, 58], amitriptyline and amoxapine, reversed social feeding in all the mutants described here. Again, this outcome was not inevitable and suggests that exaggerated threat evaluation inherent to social feeding is indeed relevant to suicide risk. Interestingly, lithium, which reduces suicidality [59], decreased social feeding in some mutants (*egl-10* and *npr-1*), enhanced it in others (*hpk-1* and *daf-7*), and was neutral in the remainder (*pdk-1* and *sad-1*). Because the PDK-1-Akt signaling pathway is a target of lithium [60], it may explain the lack of effect in *pdk-1* and *sad-1* loss-of-function mutants linked to this pathway [61]. Lithium’s aggregation promoting effects in certain mutants (*daf-7* and *hpk-1*) are notable and inspire future exploration in view of functional connections between these proteins [62]. Similarly, strain-dependent effects of haloperidol and trazodone argue for selective rather than non-specific effects of the drugs even if precise drug targets are unclear or complex. For example, completely divergent effects of haloperidol in *hpk-1* vs. *sad-1* mutants imply that the pathways contributing to social feeding can be distinguished pharmacologically, which may provide initial insights into the pharmacogenetics of drug responsiveness.

The second key finding from these studies concerns the observation that *npr-1* and *daf-7* social feeding mutants display diminished motivation to forage following food deprivation. Normally, after removal from bacteria, animals actively search locally for food (area restricted search) for 30–60 min followed later by extended forward locomotion aimed at traveling greater distances to locate food. Although *npr-1* mutants showed normal acute-stage foraging, significantly fewer *daf-7* animals were moving at 2.5 hr compared to N2. After 20 hr of food deprivation, both mutants showed greater immobility than N2 animals that were generally still very active. *npr-1* and *daf-7* mutants were not fatigued or paralyzed because they rapidly escaped sudden touch to the tail. Moreover, diminished motivation to forage was overcome by exposure to amitriptyline. We previously reported similar delayed immobility for *glr-1* mutants with loss-of-function mutations in the AMPA receptor, another suicidality-related risk gene [13]. This is noteworthy because *daf-7* signaling regulates *glr-1* expression in *C. elegans* [63], which functionally connects the suicide risk genes in this study.

**Figure 6:**
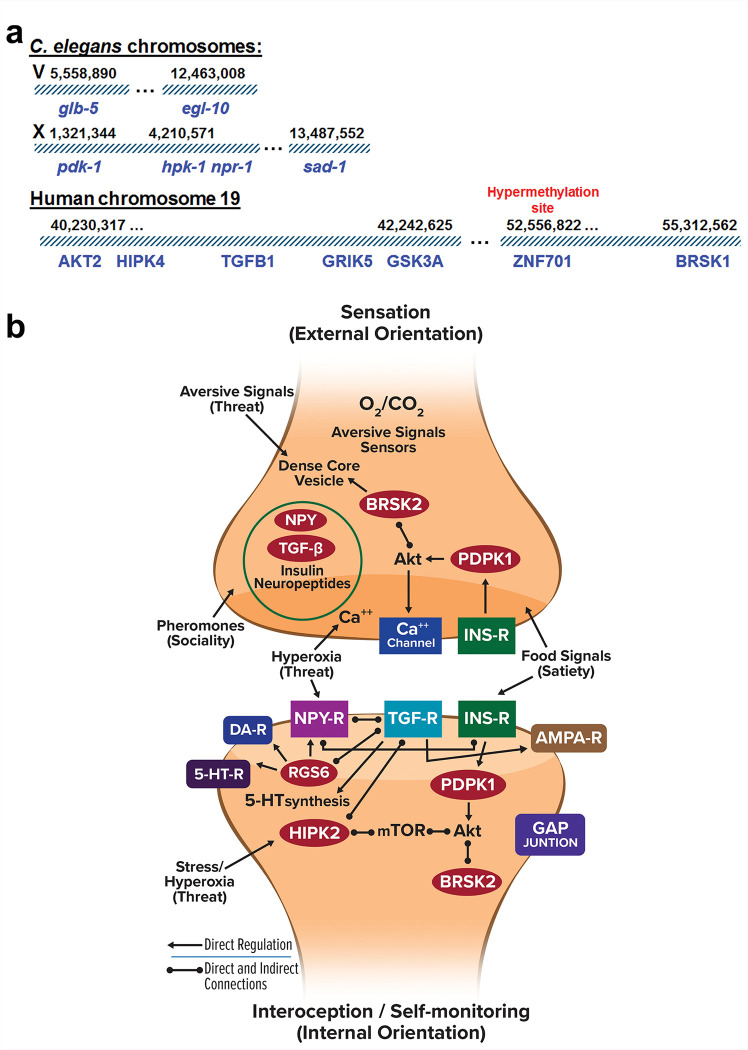
Suicide risk genes – co-localization on chromosomes and functional activities. **a)** Genes involved in social feeding in *C. elegans* co-localize on chromosomes V and X. Some of their counterparts and relevant signaling partners in humans are found together on chromosome 19q. A hypermethylation site on chromosome 19 associated with severity of suicidal ideation has been indicated. Nucleotide positions are shown above the chromosome diagrams. **b)** Using the general framework of neuronal circuits that mediate social feeding and foraging in *C. elegans*, we have represented neurons involved in sensing external signals (threats, pheromones, etc.) and interoception (monitoring internal states such as satiety). The suicide risk genes (in red ovals) are depicted in various functional pathways, and direct and indirect connections between components have been indicated with arrows and capped lines, respectively. DA-R and 5-HT-R refer to dopamine and serotonin receptors. The illustration is an over-simplification. The suicide risk genes will affect additional targets and pathways besides those shown here. Moreover, sensory and interoceptive neurons are linked by interneurons with various output targets.

The new social feeding variants characterized here are located on chromosomes V (*egl-10*) and X *(pdk-1*, *hpk-1* and *sad-1*) in *C. elegans* (Fig. 6a). Quantitative trait locus (QTL) analysis [26] suggested that besides *glb-5* (V) and *npr-1* (X) there may be additional genes on these chromosomes that affect social feeding, and our data confirm this observation. Moreover, *hpk-1* lies very close to *npr-1*, the original social feeding variant characterized. Interestingly, human counterparts to *hpk-1* (HIPK4; homeodomain interacting protein kinase 4), *daf-7* (TGFB1), *glr-1* (GRIK5) and *sad-1* (BRSK1; BR serine/threonine kinase 1) are located on chromosome 19 along with PDK-1 signaling component (AKT2) and a lithium target (GSK3A; glycogen synthase kinase 3 alpha) (Fig. 6a). Furthermore, BRSK1 is near a separate region of chromosome 19 that is hypermethylated in relation to severity of suicidal ideation and perceived stress [64]. Previously, we described syntenic blocks of risk genes for schizophrenia, bipolar disorder and major depressive disorder [65–67] possibly brought together during adaptive gene relocation [68] to enable coordinated regulation of gene expression mediating a common function. It is tempting to ascribe similar functional organization to the suicide risk genes and related components on chromosome 19q.

It was surprising to find 6 mutants that exhibited both social feeding and diminished motivation phenotypes. This behavioral overlap suggests that the genes responsible should be co-expressed in some of the neurons that regulate social feeding and foraging. As suspected, critical sensory and integrative neurons that mediate these behaviors jointly express many of the suicide risk-gene counterparts (Fig. 5a). Moreover, the human suicide risk genes are frequently co-expressed in human tissues forming networks of functionally-associated genes. These co-expression networks extend to many relevant brain areas (Table 2). For example, 8 of the 10 risk genes were co-expressed in the nucleus accumbens, a major reward/motivation center, the pontine nucleus involved in sensory integration and the claustrum that mediates sensory integration, motivation, salience and consciousness. Other brain areas where the risk genes were highly co-expressed include several nuclei of the amygdala and the bed nucleus of the stria terminalis, which mediate fear and threat assessment and the nucleus accumbens, claustrum and putamen, which contribute to goal-directed behavior and motivation more generally. These observations provide strong support for the validity of these genes as genuine risk factors for suicidality-related behavior.

How do these risk genes increase the liability for suicide including the molecular pathways involved? Previously, we [8] proposed that exaggerated threat assessment was an endophenotype for suicide because amplification of negative perceptions makes situations seem worse than they are. Moreover, sensing constant threat even when the possibility of actual harm is minimal may generate a chronic and debilitating stress response with negative psychological consequences. We further suggested that diminished motivation to engage in life activities resembled giving up or hopelessness, a known suicide risk factor [5, 7]. Consequently, genes that affect threat assessment and motivational states might be expected to impact suicidality-related behavior. Both phenotypes are based on ancient survival programs fundamental to maintaining life: sensing external threats to avoid harm and internal states such as starvation or reproductive maturation to motivate foraging and mating (Fig. 6b). Sensory neurons, interoceptive neurons and integrative interneurons mediate detection of aversive environmental signals and interoception to monitor homeostatic states such as hunger and stress.

The suicide risk genes studied here are all expressed in neurons, several are directly or indirectly related to neuropeptides, and as seen in Fig. 6b they are functionally interconnected. In general terms, the genes regulate growth (TGFB1 and PDPK1) [69, 70], coordinate the response to food signals (NPY, PDPK1 and BRSK2) [54, 71] and unfavorable growth conditions (NPY, HIPK2 and RGS6) [21, 72, 73], and affect motivation (PDPK1, TGFB1 and NPY; present studies) [39]. Regulation of the secretion of neuropeptides, including NPY and insulin, from dense core vesicles appears to be a significant common thread. In addition, crosstalk and cross-regulation between receptors for NPY, TGF-b and insulin mediate tight coordination of risk gene activity. Three of the risk genes (PDPK1, BRSK2 and HIPK2) encode protein kinases that interconnect directly and indirectly [61, 72]. Moreover, each kinase may phosphorylate multiple protein targets to simultaneously regulate different downstream pathways, thus magnifying the effect of a risk variant in these genes. Consequently, variants with small individual genetic effect sizes can have a disproportionate influence on related phenotypes along with pleiotropic effects, which could explain the genetic and mechanistic overlap between social feeding and motivation to forage. Finally, it is noteworthy that TGF-b regulates expression of tryptophan hydroxylase [74], the rate-limiting step in serotonin synthesis, given the role of serotonin in appetite, mood, depression and suicide [75].

Potential limitations of the study should be noted. First, questions may arise concerning the relevance of *C. elegans* for human suicidality-related behavior in view of the evolutionary distance and simplicity of this model organism. However, suicide risk genes identified in genome-wide surveys are highly conserved during evolution with 88% expressed in *C. elegans* [13]. Moreover, the endophenotypes explored in this study represent fundamental biological functions such as threat assessment, motivation to find food, and internal state monitoring that are required for the survival of all species. Secondly, the drugs used to reverse the phenotypes described here have complex pharmacological actions, e.g., amitriptyline, chlorpromazine and cyproheptadine also inhibit calmodulin. However, it is encouraging that so many drugs with relevance for depression and suicide had restorative effects in our behavioral assays.

To briefly recap, suicide risk genes promote social feeding (exaggerated threat assessment) in *C. elegans* that was corrected with drugs such as antidepressants and clozapine, which effectively reduce suicidal behavior [57–59]. Furthermore, two established social feeding mutants showed diminished motivation to search for food that was reversed with amitriptyline similar to other strains described previously [13] with mutations in suicide risk genes. How does this relate to the experiences of someone contemplating suicide? The sensory and interoception systems affected by suicide risk-gene counterparts in *C. elegans* detect external threats, internal homeostatic states and mediate the drive to optimize living conditions (e.g., by searching for food or locating a safer environment). There are intriguing parallels to individuals with suicidal thoughts. Their thinking may focus on overestimated external threats and slights together with distress from internal mental states such as hopelessness while lacking the motivation to change the situation rather than choosing to end it. Under this scenario, suicidal ideation occurs when existential threats seem overwhelming and motivational systems fail to inspire positive actions and hope. By exploring the function of suicide risk genes in *C. elegans*, we discovered that genes predisposing a person to take their life normally play a critical role in survival.

## Figures and Tables

**Table 1.** *C. elegans* strains with mutations in putative suicidality-related risk genes

Strain	*C. elegans* gene (allele)	Human gene	Diminished motivation phenotype	Corrected with drugs*	Social feeding phenotype	Corrected with drugs^&^
CB271	*unc-40(e271)*	DCC	+	AD, C/L, Li	−	
CB1372	*daf-7(e1372)*	TGFB	+	AD, C/L	+	AD, C/L, Li^#^
CB5380	*fox-1(e2643)*	RBFOX1	+	AD, C/L	−	
CX4148	*npr-1(ky13)*	NPY1R	+	AD, C/L, Li	+	AD, C/L, Li
CX5156	*sad-1(ky289)*	BRSK2/STK33	+	AD, C/L, Li	+	AD, C/L
DA609	*npr-1(ad609)*	NPY1R	+	AD, C/L, Li	+	AD, C/L, Li
DA650	*npr-1*	NPY1R	+	AD, C/L, Li	+	AD, C/L, Li
DA2250	*mgl-2(tm355);* *mgl-1(tm1811)*	GRM5	−		−	
EK273	*hpk-1(pk1393)*	HIPK2/3	+	AD, C/L, Li	+	AD, C/L, Li^#^
JC1970	*tbx-2(ut180)*	TBX20	+	AD, C/L, Li^#^	−	
JT9609	*pdk-1(sa680)*	PDPK1	+	AD, C/L	+	AD, C/L
JY359	*lim-4(yz12)*	LHX6	−		−	
KP4	*glr-1(n2461)*	GRIA1	+	AD, C^†^	−	
MT7562	*sqv-7(n2839)*	SLC35D1	−		−	
MT8504	*egl-10(md176)*	RGS6	+	AD, C/L, Li	+	AD, C/L, Li
OH161	*ttx-3(ot22)*	LHX6	+	AD, C/L, Li	−	
QL300	*tph-1(n4622);* *daf-7(ok3125)*	TPH1; TGFB1	+	AD, C/L	+	AD, C/L, Li^#^
RB1231	*pde-4(ok1290)*	PDE4B	−		−	
RB1360	*igcm-2(ok1527)*	IGSF9B	−		−
RB1367	*cpx-1(ok1552)*	CPLX1	+	AD, C/L, Li	−
RB1668	*npr-19(ok2068)*	CNR1	−		−
RB1888	*gly-10(ok2439)*	GALNT10	+	AD, C/L, Li	−

(+) Refers to presence of the phenotype whereas (-) indicates absence of it.

* Drugs that corrected the diminished motivation phenotype as determined in previous studies [13] or as reported here.

& Results of this study.

# Lithium significantly exacerbated the phenotype in this strain.

† Loxapine was not tested with the *glr-1* mutant.

Drug abbreviations: AD antidepressants (amitriptyline and amoxapine), C clozapine, L loxapine, Li lithium

**Table 2:** Human brain areas co-expressing suicide risk genes

Brain area	Function	Genes co-expressed
Nucleus accumbens right	Reward/aversion, motivation, addiction	GRIA1 PDPK1 TGFB1 STK33 HIPK2 BRSK2 NPY1R NPY
Claustrum left	Integrates sensory information; salience; consciousness and volition	GRIA1 HIPK3 PDPK1 TGFB1 HIPK2 BRSK2 NPY1R NPY
Pontine nuclei left	Relay hub; integration; motor function	HIPK3 PDPK1 TGFB1 STK33 HIPK2 RGS6 BRSK2 NPY1R
Basomedial nucleus left	Regulation of fear, anxiety & stress	GRIA1 HIPK3 PDPK1 TGFB1 HIPK2 NPY1R NPY
Lateral nucleus amygdala left	Sensory gateway, fear/emotional learning, threat evaluation	GRIA1 PDPK1 TGFB1 STK33 BRSK2 NPY1R NPY
Lateral nucleus amygdala right	Sensory gateway; fear/emotional learning; threat evaluation	GRIA1 HIPK3 PDPK1 TGFB1 BRSK2 NPY1R NPY
Precuneus left	Self-monitoring/awareness; information integration/retrieval	GRIA1 PDPK1 STK33 HIPK2 RGS6 BRSK2 NPY
Anterior orbital gyrus right	Sensory integration; processing rewards/aversive stimuli	GRIA1 PDPK1 TGFB1 HIPK2 BRSK2 NPY
CA4 field left	Memory function; mood regulation; sleep	GRIA1 HIPK3 PDPK1 BRSK2 NPY1R NPY
CA4 field right	Memory function; mood regulation; sleep	GRIA1 PDPK1 TGFB1 HIPK2 BRSK2 NPY
Claustrum right	Integrates sensory information; salience; consciousness and volition	GRIA1 HIPK3 PDPK1 TGFB1 BRSK2 NPY1R
Caudal group intralaminar nuclei left	Pain/nociception; motor control; arousal	PDPK1 TGFB1 STK33 HIPK2 BRSK2 NPY1R
Piriform cortex left	Perception of smells; social behavior	GRIA1 HIPK3 TGFB1 HIPK2 NPY1R NPY
Basolateral nucleus left	Fear learning; integrates sensory and emotional information; motivation	GRIA1 HIPK3 PDPK1 HIPK2 BRSK2 NPY1R
Putamen left	Motor regulation; goal-directed behavior	GRIA1 HIPK3 PDPK1 STK33 NPY1R NPY
Spinal trigeminal nucleus	Relays pain and temperature sensations	HIPK3 PDPK1 TGFB1 STK33 BRSK2 NPY1R
Fusiform gyrus left	Processing visual information; word recognition	GRIA1 PDPK1 RGS6 BRSK2 NPY1R NPY
Bed nucleus stria terminalis	Regulates stress, anxiety & fear. Also motivation and threat evaluation	GRIA1 HIPK3 TGFB1 STK33 NPY1R NPY
Inferior frontal gyrus orbital part left	Cognitive function; language processing; sociality	GRIA1 PDPK1 RGS6 BRSK2 NPY1R NPY
Inferior colliculus left	Integrates auditory signals and sensory information; arousal	GRIA1 HIPK3 STK33 HIPK2 BRSK2 NPY1R

* Gene expression data were obtained from the Allen Brain Atlas website [50]. Expression levels were detected with the following human gene probes: GRIA1 - CUST_13840_P1416261804; HIPK3 - A_23_P422809; PDPK1 - A_24_P222599; TGFB1 - CUST_651_P1416408490; STK33 - A_23_P127915; HIPK2 - A_32_P738061; RGS6 - A_32_P126557; BRSK2 - A_24_P326708; NPY1R - CUST_10388_P1416261804; NPY - A_23_P256470. Suicide risk genes were considered to be co-expressed if their z-scores were >1.0 in the brain area examined. We only showed co-expression for those cases where 6 or more genes had z-scores above 1 in a particular brain area noted in the left column of the Table. Regional brain functions were obtained from : https://www.sciencedirect.com/topics/neuroscience. Functional activities most relevant to the *C. elegans* protophenotypes have been highlighted in blue font.
